# Accumulation of Anthocyanins: An Adaptation Strategy of *Mikania micrantha* to Low Temperature in Winter

**DOI:** 10.3389/fpls.2019.01049

**Published:** 2019-08-29

**Authors:** Qilei Zhang, Junjie Zhai, Ling Shao, Wei Lin, Changlian Peng

**Affiliations:** ^1^Guangzhou Key Laboratory of Subtropical Biodiversity and Biomonitoring, Guangdong Provincial Key Laboratory of Biotechnology for Plant Development, School of Life Sciences, South China Normal University, Guangzhou, China; ^2^College of Life Science, Zhao Qing University, Zhaoqing, China

**Keywords:** anthocyanins, antioxidant activity, gas exchange, *Mikania micrantha*, winter

## Abstract

The accumulation of anthocyanins in leaves and stems of *Mikania micrantha* improves its adaptability to low-temperature environments during winter in areas where this species is invasive. The accumulation of anthocyanins in *M. micrantha* causes the plants to exhibit red coloration when encountering low-temperature environments during winter. Many studies have reported that the accumulation of anthocyanins near the plant surface filters light and improves photoprotection. However, the results of this study showed that the main role of anthocyanins accumulation in *M. micrantha* during winter was to increase both antioxidant capability and tolerance to low temperature. The results showed that the anthocyanin contents were significantly higher in red leaves and stems than in green leaves and stems, with more than 60-fold greater content in red leaves than in green leaves. In addition, the total antioxidant capability was significantly greater in red leaves and stems than in green leaves and stems. After 4°C treatment for 12 h, a large amount of reactive oxygen species accumulated in green leaves and stems, and the maximum photochemical efficiency decreased significantly. Compared with that of the green leaves, the net photosynthetic rate of red leaves was significantly higher. The biomass statistics revealed that the dry matter accumulation of *M. micrantha* plants with relatively large amounts of anthocyanins was significantly greater than that of plants with relatively low anthocyanin levels during the same period. Our results suggest that the accumulation of anthocyanins during winter is an adaptation strategy of *M. micrantha* to low winter temperatures.

## Introduction

Low-temperature stress is considered the main cause of plant growth constraints and crop yield reductions (Jin et al., 2017; Sun et al., 2018). Low-temperature stress can damage the photosynthetic apparatus and reduce photosynthetic efficiency (Yang et al., 2016), induce oxidative stress, and lead to the generation of reactive oxygen species (ROS) in tissues, reduce the fluidity of membranes (Gusta and Wisniewski, 2013), induce the degradation of proteins (Ruelland et al., 2009), and cause the inhibition of the activity of antioxidant enzymes (Baek and Skinner, 2003). Higher plants have developed many strategies to reduce the damage caused by low-temperature stress (Chinnusamy et al., 2007). Under low-temperature conditions, some plant species present tolerance mechanisms in a genotype-, organ-, or environment-specific manner *via* various complex networks of metabolic pathways (Zhang et al., 2014). Plants can improve their membrane fluidity by increasing the amount of unsaturated fatty acids. The accumulation of nonenzymatic substances (such as anthocyanins, phenylpropanoids, and terpenoids) increases antioxidant capabilities, thereby reducing oxidative stress caused by low temperature (Arnholdt-Schmitt et al., 2006; Sivankalyani et al., 2016). Moreover, anthocyanin accumulate increased significantly in flowers, seeds, fruits, and vegetative tissues in response to low-temperature stress (Allan et al., 2008; Liu et al., 2018).

Anthocyanins are natural, water-soluble pigments that belong to the flavonoid family of plant secondary metabolites. Anthocyanins are ubiquitous in flowers, fruits, stems, and leaves of plants (Tanaka and Ohmiya, 2008). The function of anthocyanin in plants involves protection against various biotic and abiotic stresses (Ahmed et al., 2014). Various environmental factors such as nutrition, drought, light, and temperature affect the accumulation of anthocyanins in many plant species (Allan et al., 2008). In the anthocyanin biosynthetic pathway, chalcone synthase (CHS), chalcone isomerase (CHI), flavanone 3-hydroxylase (F3H), dihydroflavonol 4-reductase (DFR), and anthocyanidin synthase (ANS) are the main enzymes controlling the synthesis of anthocyanins (Tanaka et al., 2008; Liu et al., 2018). Anthocyanins can protect plants against UV light, pathogens, and low temperature (Field et al., 2001; Sivankalyani et al., 2016). Low-temperature conditions can cause oxidative stress in plants. Anthocyanins exhibit antioxidant activity and can improve the antioxidant capability of plants (Pojer et al., 2013). Under low-temperature stress, the expression of genes related to anthocyanin synthesis in plants significantly increases, and anthocyanin accumulation increases, which improves plant tolerance to low temperature (Lo Piero et al., 2005; Naing et al., 2018). In woody plants, the accumulation of anthocyanin in young leaves during winter can reduce oxidative damage and increase photosynthetic rates (Zhu et al., 2018).

*Mikania micrantha* HBK belongs to the *Asteraceae* family and is a fast-growing and rapidly reproducing perennial creeping vine indigenous to Central and South America. *M. micrantha* is among the top 100 invasive plants worldwide (Holm et al., 1977; Yang et al., 2005). It is currently considered an invasive species in Southeast Asia and the Pacific region, including in South China (Wang et al., 2016). Substantial damage to agriculture, forestry, and ecological balance has occurred because of the rapid spread of *M. micrantha* in South China (Zhang et al., 2004). As a creeping vine, *M. micrantha* covers trees and grasses and ultimately kills them by depriving them of their light source (Siwakoti, 2007). *M. micrantha* is a thermophilic and sun-loving plant species. However, in South China, where *M. micrantha* is invasive, it also encounters a low-temperature environment (below 15°C) during winter (Yan et al., 2013); in the field, most of the *M. micrantha* plants had accumulated anthocyanins and turned red during the winter. This study aimed to elucidate the role of anthocyanins in the invasive capability of *M. micrantha* during winter.

## Materials and Methods

### Plant Materials

In September, *M. micrantha* was planted *via* vegetative reproduction in the biological garden of South China Normal University, Guangzhou, China. Branches of *M. micrantha* without leaves were cut into segments that had two stem nodes; the segments were subsequently planted into soft soil that was tilled in accordance with the cutting method. The planting area was maintained with routine weeding and watering. In December, the same batch of *M. micrantha* planted in September exhibited different colors, with some plants having turned red and some remaining green. The stems and leaves of different-colored plants were selected as research materials. Three to 15 biological replicates were incorporated in this study.

### Pigment Estimation

Anthocyanins were extracted from 0.05 g of fresh leaves or stems in 2 ml of a methanol:HCl (99:1, v/v) solution at 4°C in the dark for 24 h (Manetas et al., 2003; Hughes et al., 2007). Chlorophyll (Chl) was removed from the extracts by the addition of 2 ml of chloroform and 1 ml of purified water. After the solution was mixed, the anthocyanins dissolved in the upper water-methanol phase, and the Chl dissolved in the lower chloroform phase. The absorption spectra of the anthocyanin extracts at 420–700 nm were recorded against that of methanol:HCl (99:1, v/v) as a blank using a UV-Vis 2450 spectrophotometer (Shimadzu, Tokyo, Japan). The absorbance at 530 nm was used to calculate the anthocyanin content, and different concentrations of cyanidin-3-glucoside were used as standards.

The Chl was extracted from 0.05 g of fresh leaves in 4 ml of an 80% acetone solution at 4°C in the dark for 24 h. The absorbance of the Chl extracts was recorded using a UV-Vis 2450 spectrophotometer (Shimadzu, Tokyo, Japan), and the Chl content was calculated according to the methods of Wellburn (1994).

Total phenols were extracted from 0.05 g of fresh leaves or stems in 2 ml of a 95% methanol solution at 4°C in the dark for 24 h. The total phenol content was measured according to the Folin–Ciocalteu method as described by Ainsworth and Gillespie (2007). First, 1 ml of 10% Folin–Ciocalteu reagent and 2 ml of 0.7-M Na_2_CO_3_ were added to a 0.5-ml sample. The absorbance of the mixture was subsequently measured at 765 nm (*UV-2450*, Shimadzu, Kyoto, Japan) after it was mixed for 5 min. Different concentrations of gallic acid were used as standards.

Flavonoids were extracted from 0.05 g of fresh leaves or stems in the same way as the total phenols were and measured according to the method described by Heimler et al. (2005), with slight modifications. One hundred fifty microliters of the sample, 1.85 ml of deionized water, 0.2 ml of 5% NaNO_2_, 0.3 ml of 10% AlCl_3_ (freshly prepared), and 1 ml of 1-M NaOH solution were added to a 5-ml centrifuge tube. The absorbance of the mixture was measured at 510 nm (UV-2450, Shimadzu, Kyoto, Japan) after it was mixed for 5 min. Different concentrations of catechin were used standards.

### Total Antioxidant Capability Determination

The total antioxidant capability was evaluated by a 1,1-diphenyl-2-picrylhydrazyl (DPPH) test according to the method of Saha et al. (2008), with slight modifications. At 4°C in the dark for 24 h, 0.05 g of fresh leaves or stems were placed in 2 ml of a 95% methanol solution. For 5 min in the dark, 0.1 ml of the sample was then added to 2.9 ml of 120-μM DPPH (dissolved in 95% methanol). The mixture was subsequently measured at 517 nm (UV-2450, Shimadzu, Kyoto, Japan). Different concentrations of DPPH were used as standards, and the free radical-scavenging capability of the mixture was calculated according to the absorbance of the mixture at 517 nm.

### Gene Expression Analysis

Total RNA from the leaf samples was extracted using TRIzol reagent (Invitrogen, MA, USA) according to the manufacturer’s instructions. Complementary DNA was synthesized using TopScript™ RT DryMIX (dT18) (Enzynomic, Daejeon, Korea) according to the manufacturer’s instructions. Quantitative reverse transcription polymerase chain reaction analysis was performed using a SYBR Premix Ex Taq^™^ II Kit (Takara) in conjunction with a Bio-Rad CFX96 Real-Time PCR System (CFX96, Bio-Rad, USA). *Actin* was used as a reference gene; the primer pairs for which were 5′-TGAAATACCCCATTGAGCATGG-3′ (forward) and 5′-GAATCCAGTACAATACCTGTGGTAG-3′ (reverse). The relative expression of the anthocyanin biosynthetic pathway genes *chalcone synthase* (*CHS*), *chalcone isomerase* (*CHI*), *flavanone 3-hydroxylase* (*F3H*), *dihydroflavonol 4-reductase* (DFR), and *anthocyanidin synthase* (*ANS*) was measured. The primers for real-time PCR were as follows: 5′-ACATGCCTGGTGCAGATTACCA-3′ (forward) and 5′-AAGTGGGAATCGGAAGGTCCAC-3′ (reverse) for *CHS*; 5′-GGAGGCGGTTCTGGAATCTATC-3′ (forward) and 5′-TCGTCCTTGTTCTTCATCATTAGC-3′ (reverse) for *CHI*; 5′-TTGCAGGCCAGGCCCATT-3′ (forward) and 5′-TGCAAGATTGGAGGGAGATTGT-3′ (reverse) for *F3H*; 5′-AGCTTTGATGAAGCCATTSAAGGTTGC-3′ (forward) and 5′-TTCTTCACTGTCTTGGCTTT-3′ (reverse) for *DRF*; and 5′-TCAGCCGGTTGAAGAGAAGGAG-3′ (forward) and 5′-GAGGGCCAAATGGTCAAATCACGT-3′ (reverse) for *ANS*. The 10-μl reaction mixture consisted of 5 μl of 2× Premix Ex Taq II, 0.4 μl of the forward primer (10 μM), 0.4 μl of the reverse primer (10 μM), 0.5 μl of complementary DNA template (< 100 ng), and 3.7 μl of ddH_2_O. The cycle conditions were as follows: initial denaturation at 95°C for 30 s; 40 cycles at 95°C for 5 s and 64°C for 34 s; 1 cycle at 95°C for 15 s (for recording a melting curve); and one cycle at 64°C for 1 min. The relative gene transcript levels were calculated using the 2^-ΔΔ^ *^C^* ^T^ method (Livak and Schmittgen, 2001).

### Rubisco Protein

Fresh leaves (0.1 g) were weighed, placed in a precooled mortar (with a small amount of inert quartz) on ice, and homogenized in 1.5 ml of 60-mM Tris-HCl (pH 7.8) buffer containing 5% (w/v) polyvinylpyrrolidone, 0.1% (w/v) NaCl, and 2% (v/v) glycerol. The grinding fluid was poured into a 2-ml centrifuge tube, which was subsequently centrifuged at 13,000×*g* for 10 min at 4°C; the total protein was contained within the supernatant. To analyze the Rubisco content, 0.1 ml of the supernatant was added to an equal volume of protein loading buffer, which was then incubated at 100°C for 5 min after it was mixed. The 12.5% sodium dodecyl sulfate polyacrylamide gel electrophoresis (SDS-PAGE) gel was made according to the methods of Zhang et al. (2016). Each 20-μl sample was loaded in the wells of the gel, and the Rubisco protein was separated by SDS-PAGE with a Mini-PROTEAN 3 system (Bio-Rad, USA). The large and small subunits of Rubisco were recognized according to their molecular mass and the included marker stained by Coomassie Brilliant Blue R-250. The Rubisco large subunit (LS) was identified using rabbit antibodies against the Rubisco protein. After being separated on a 12.5% SDS-PAGE gel, the total proteins were blotted onto a polyvinylidene fluoride (PVDF) membrane, and then, the PVDF membrane was stained by Ponceau S for 10 min. The results stained by Ponceau S were recorded with digital camera, and then, the PVDF membrane was washed three times (5 min each) in Tris-buffered saline with Tween 20 (TBST) buffer [8.8 g of NaCl, 20 ml of 1 M Tris-HCl (pH = 8.0), and 0.5 ml of Tween 20 added to 1 l of water]. The PVDF membrane containing the blots was subsequently preincubated in TBST buffer that contained 5% nonfat powdered milk for 1.5 h at room temperature. The preincubated PVDF membrane was washed three times (3 min each) in TBST buffer and then incubated with anti-Rubisco antibody (1:1,000 dilution) (Bioss, Beijing, China) for 1 h at room temperature. The incubated PVDF membrane was washed three times (5 min each) in TBST buffer and then incubated for 50 min with goat anti-rabbit HRP-conjugated secondary antibody (1:3,000 dilution) at room temperature, after which the membrane was washed another three times (5 min each) in TBST buffer. The anti-Rubisco labeling of the blots was visualized *via* chemiluminescent HRP substrate (Millipore, Billerica, USA) and imaged with a Tanon 5200 system (Tanon, Shanghai, China).

### Chlorophyll Fluorescence Determination

A Chl fluorescence imaging system (Technologica, UK) was used to measure Chl fluorescence. Leaves and stems were removed from *M. micrantha* plants growing at 4°C for 12 h, and a room temperature treatment was used as a control (CK). The leaves and stems were then placed in the dark for 30 min. The minimum fluorescence (*F*_0_) and the maximum fluorescence (*F*_m_) of the leaves and stems were measured using a 6,000 μmol m^–2^ s^–1^ saturating pulse. The maximum photochemical efficiency (*F*_v_/*F*_m_) of photosystem II (PSII) was calculated as *F*_v_/*F*_m_ = (*F*_m_ – *F*_0_)/*F*_m_ (Oxborough and Baker, 1997).

### Gas Exchange

Gas exchange was measured using an LI-6800 Portable Photosynthesis System (LI-COR, Inc., USA) in the morning (9:00–11:00) on sunny days in accordance with the method described by Mega et al. (2019), with moderate adjustments. The gas exchange of fully expanded mature *M. micrantha* leaves was measured at an irradiance of 800 μmol m^–2^ s^–1^. The ratio of the red and blue light of the irradiance in the leaf measurement chamber was set to 9:1, the mean relative air humidity was 45%, and the corresponding mean temperature was 20°C. The net photosynthetic rate (*P*_n_), intercellular CO_2_ concentration (*C*_i_), stomatal conductance (*G*_s_), and transpiration rate (*T*_r_) were recorded when they were relatively stable.

### Stomatal Observations

The stomata were measured in accordance with the method described by Zhang et al. (2016), with slight modifications. The leaves were cut into 2 × 2-mm fragments and put into a fixing solution that consisted of 2.5% glutaraldehyde and 2% polyformaldehyde at 4°C for more than 12 h. The leaf fragments were then dehydrated with an ethanol concentration gradient (30–100%) every 20 min. The dehydrated leaf fragments were critical-point dried with CO_2_ and then sputtered with a 30-nm gold layer. The stomata were subsequently observed *via* a scanning electron microscope (SEM) (Q25, FEI, USA).

### Hydrogen Peroxide and Superoxide Histochemical Staining

3,3’-Diaminobenzidine (DAB) and nitroblue tetrazolium (NBT) staining were performed to detect the accumulation of hydrogen peroxide and superoxide, respectively (Liu et al., 2007). After, they were treated at 4°C for 12 h; leaves and stems were dipped quickly into potassium phosphate buffer (50 mM, pH 7.0) that contained 0.5 mg ml^−1^ DAB and potassium phosphate buffer (50 mM, pH 6.4) that contained 1 mg ml^−1^ NBT under vacuum for 30 min, after which they were incubated at room temperature for 8 or 12 h (stained with DAB or NBT, respectively) in the dark. Subsequently, methanol:HCl (99:1, v/v) and 80% acetone solutions were used to clarify anthocyanins and Chl in the stained plant samples, respectively. Images of the stained plant samples were taken with a digital camera.

### Biomass Statistics

The red and green branches of *M. micrantha* were marked. After 10 days, the marked branches were removed, heated at 105°C for 20 min, and then, dried at 80°C for 72 h. The *M. micrantha* dry matter that accumulated during 10 days was determined *via* an electronic balance. The diameter of *M. micrantha* was measured using a Vernier caliper.

### Statistical Analysis

Statistical significance was determined by one-way analysis of variance (ANOVA) followed by Duncan’s *post hoc* test or Student’s *t*-test using SPSS Statistics 19.0 (IBM, NY, USA). The means were considered significantly different at *P* < 0.05. SigmaPlot 12.5 (Systat Software Inc., USA) was used to conduct linear regression analysis and to plot the data. All the data are shown as the means ± standard errors (SEs).

## Results

### Accumulation of Anthocyanins

**Figure 1:**
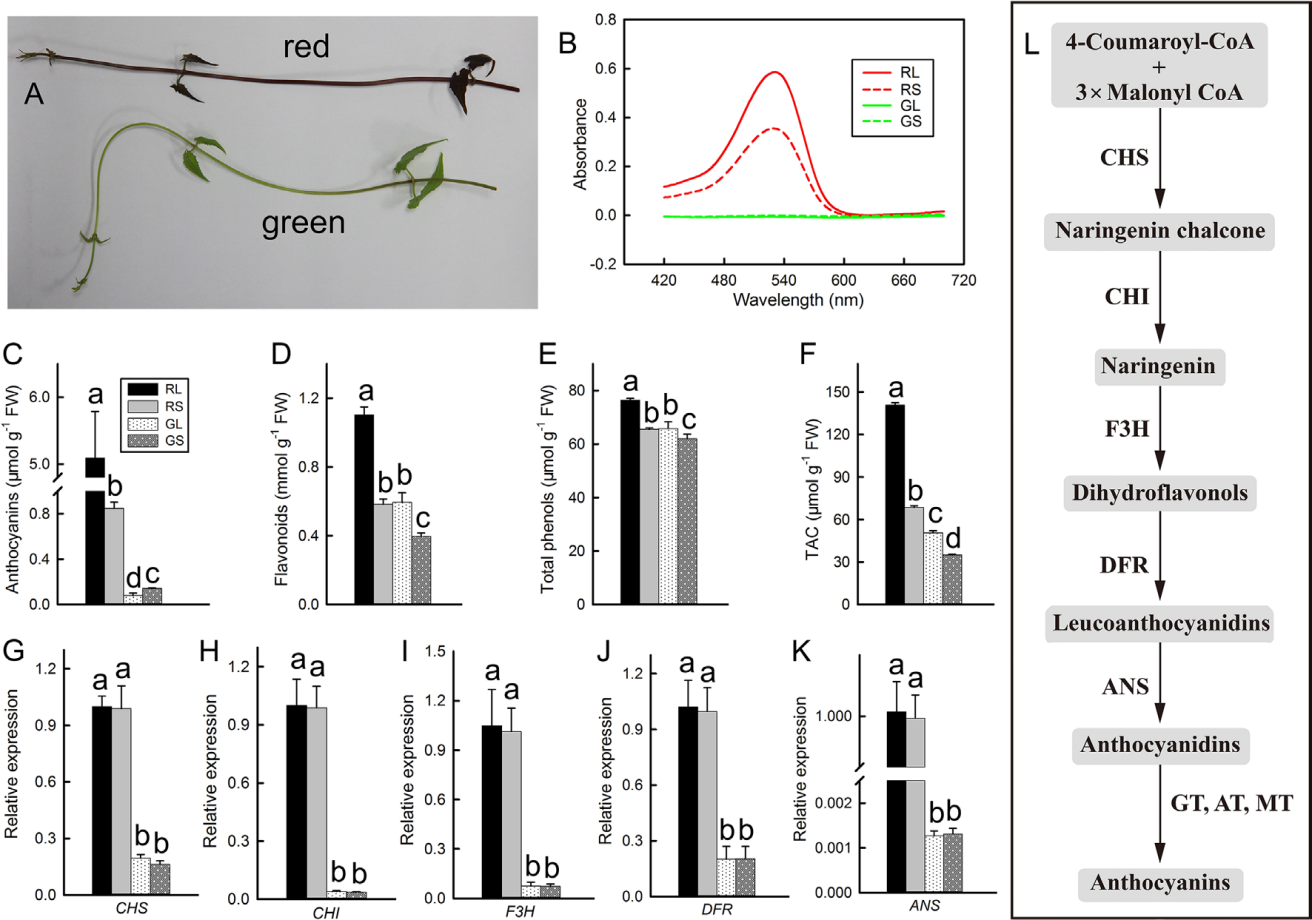
Phenotypes of different-colored *M. micrantha* **(A)**. Absorbance spectra of anthocyanin extracts from red leaves (RL), red stems (RS), green leaves (GL), and green stems (GS) of *M. micrantha* (n = 5) **(B)**. Contents of anthocyanins **(C)**, flavonoids **(D)**, and total phenols **(E)** and the total antioxidant capability **(F)** in the RL, RS, GL, and GS of *M. micrantha* (n = 8). Relative expression of the anthocyanin biosynthetic pathway genes *chalcone synthase* (*CHS*), *chalcone isomerase* (*CHI*), *flavanone 3-hydroxylase* (*F3H*), *dihydroflavonol 4-reductase* (DFR), and *anthocyanidin synthase* (*ANS*) **(G–K**, respectively**)** in the RL, RS, GL, and GS of *M. micrantha* (n = 6). The *actin* gene was used for normalization. The schematic picture of anthocyanin biosynthesis **(L)**. The error bars represent the standard errors (SEs) of five to eight biological replicates. One-way analysis of variance (ANOVA) was used to evaluate the statistical significance; the bars with different letters (a, b, c, d) indicate significant differences between the means (least significant difference, *P* < 0.05).

During winter, *M. micrantha* exhibited different colors, with some plants turning red and some remaining green (**Figure 1A**). The spectral absorption peaks of the anthocyanin extracts from the leaves and stems occurred at 530 nm (**Figure 1B**), demonstrating the presence of anthocyanins. The anthocyanin contents in red leaves and stems were significantly greater than those in green leaves and stems, and the highest anthocyanin content was detected in red leaves (**Figure 1C**). The anthocyanin content in red leaves was more than 60 times that in green leaves, and the content in red stems was more than six times that in green stems. The contents of flavonoids and total phenols were the same: these contents were greatest in red leaves, lowest in green stems, and moderate in both red stems and green leaves, and there was no significant difference in flavonoid or total phenol contents between red stems and green leaves (**Figures 1D, E**). The total antioxidant capability of red leaves and stems was significantly greater than that of green leaves and stems, and the total antioxidant capability of the leaves was significantly greater than that of the stems (**Figure 1F**). The expression of genes related to anthocyanin synthesis (*CHS*, *CHI*, *F3H*, *DFR*, and *ANS*) in red leaves and stems was significantly greater than that in green leaves and stems, and the expression of the downstream gene *ANS* in red leaves and stems was nearly 1,000 times greater than that in green leaves and stems. These results indicated that anthocyanins were synthesized vigorously in red leaves (**Figures 1G–K**). A schematic of anthocyanin biosynthesis is shown in **Figure 1L**.

### Chl Fluorescence and Localization of Reactive Oxygen Species

**Figure 2:**
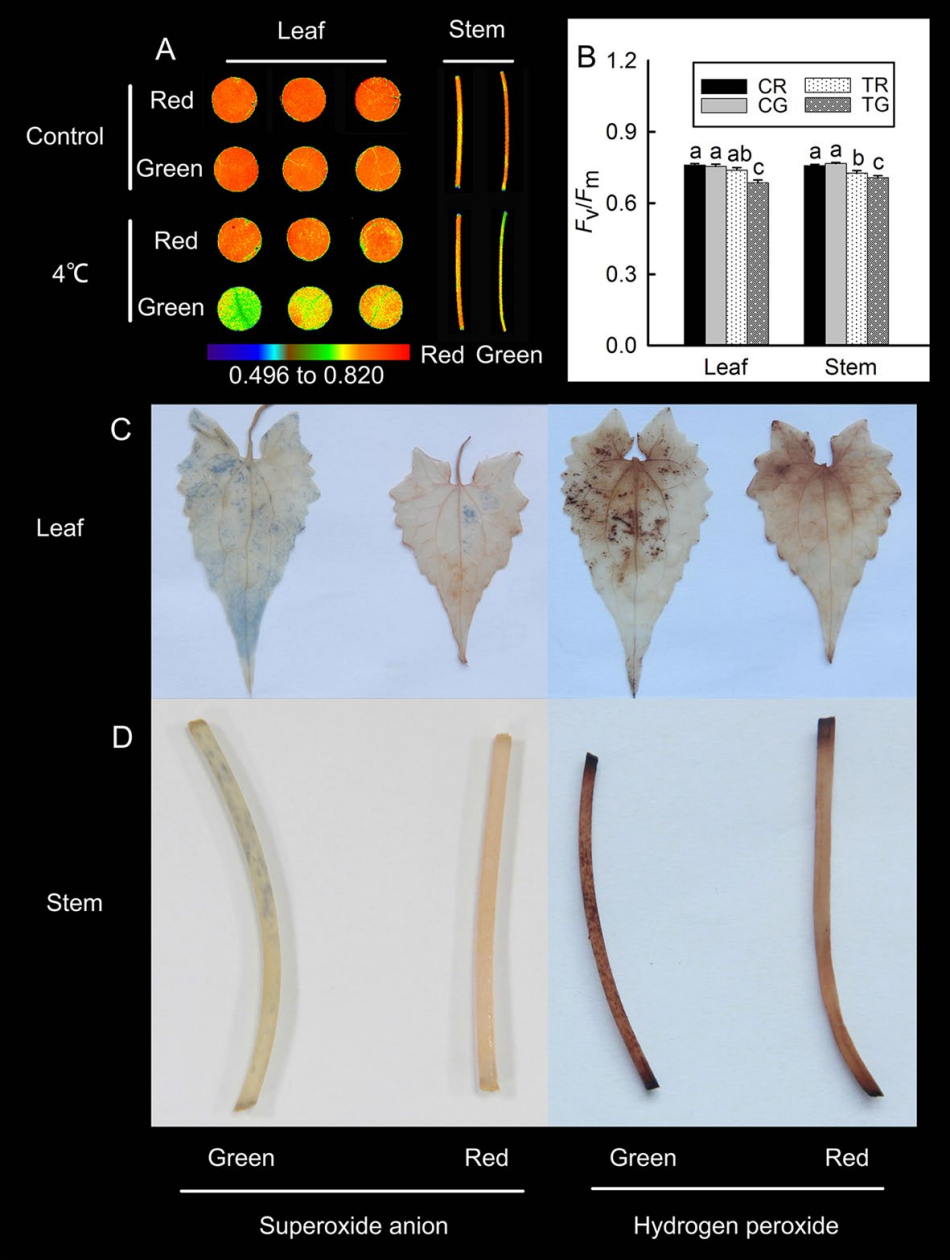
Changes in the maximum photochemical efficiency (*F*_v_/*F*_m_) of the red leaves (RL), red stems (RS), green leaves (GL), and green stems (GS) of *M. micrantha* after 4°C treatment **(A)**, the *F*_v_/*F*_m_ value of red and green in control (CR, CG) and 4°C treatment (TR, TG) **(B)**. Tissue localization of superoxide anions and hydrogen peroxide in the RL, RS, GL, and GS of *M. micrantha* was observed after 4°C treatment **(C**, **D)**. The error bars represent the standard errors (SEs) of three biological replicates. One-way analysis of variance (ANOVA) was used to evaluate the statistical significance; the bars with different letters (a, b, c, d) indicate significant differences between the means (least significant difference LSD, *P* < 0.05).

According to the previously discussed results, *M. micrantha* exhibited different colors under low temperatures during winter: red and green. The accumulation of anthocyanins in leaves is associated with resistance to low-temperature stress (Ahmed et al., 2014). To detect differences in low-temperature tolerance between the red and green *M. micrantha* plants, we treated the two different-colored *M. micrantha* plants with low temperature (4°C). The value of *F*_v_/*F*_m_ in plants will decrease significantly after stress. In this study, the Chl fluorescence results revealed no significant differences in the *F*_v_/*F*_m_ values between the red and green tissue in the control group. Compared with the control group, the value of *F*_v_/*F*_m_ in red leaves had no significant decline, and it decreased significantly in red stems after 4°C treatment. The value of *F*_v_/*F*_m_ in green leaves and stems decreased significantly after 4°C treatment (**Figures 2A, B**). These results suggest that anthocyanins can improve plant tolerance to low temperature. DAB and NBT were used to detect the localization of hydrogen peroxide and superoxide anions in the leaves and stems of *M. micrantha* after 4°C treatment, respectively. The accumulation of hydrogen peroxide and superoxide anions was relatively low in red leaves and stems, but a large amount of hydrogen peroxide and superoxide anions accumulated in green leaves and stems (**Figures 2C, D**).

### Gas Exchange Parameters and Biomass

**Figure 3:**
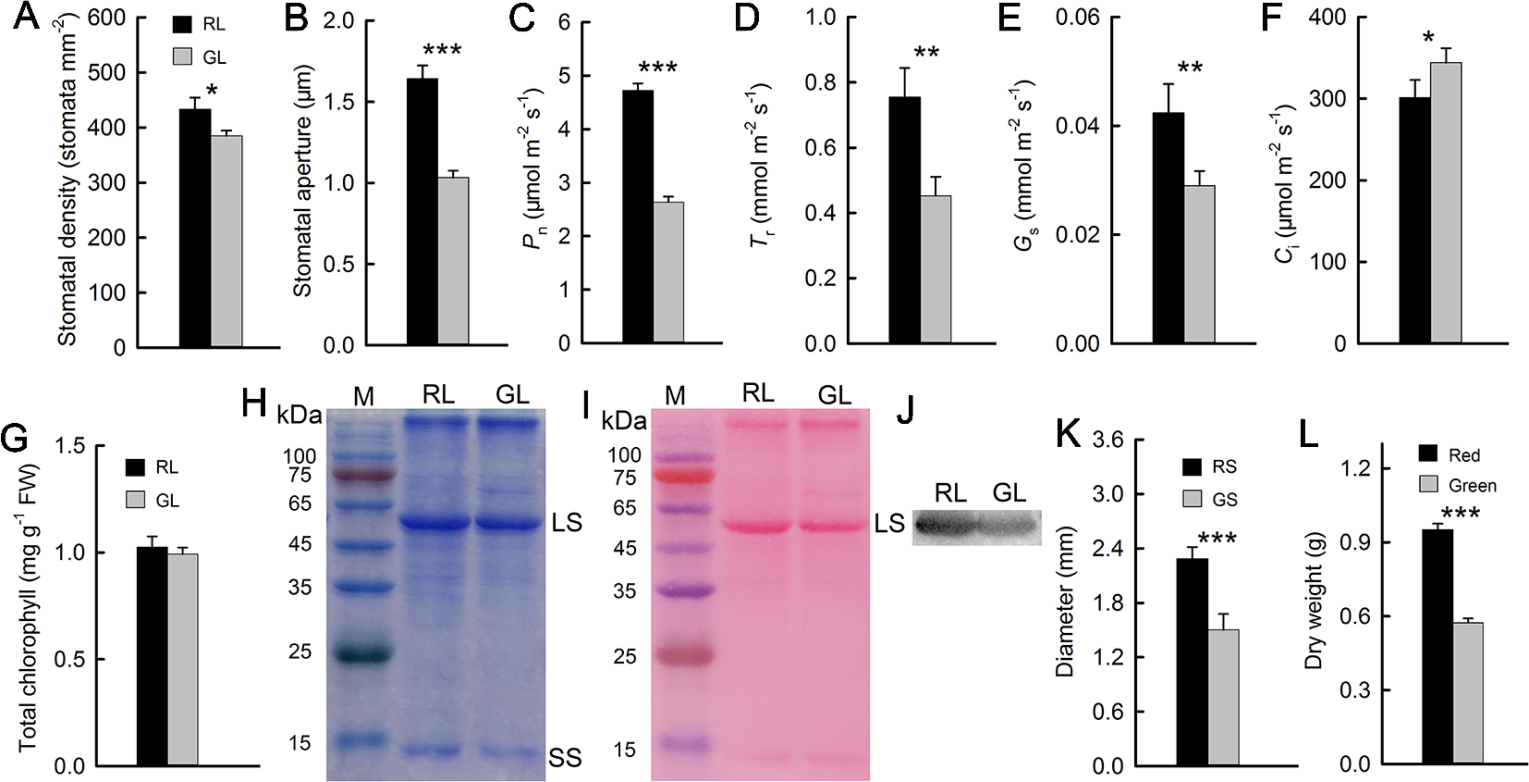
Stomatal density and aperture of red (RL) and green leaves (GL) **(A**, **B)**. Gas exchange parameters of red and green leaves, including the net photosynthetic rate (*P*_n_) **(C)**, transpiration rate (*T*_r_) **(D)**, stomatal conductance (*G*_s_) **(E)**, and intercellular CO_2_ concentration (*C*_i_) **(F)** (n = 10). Chlorophyll (Chl) content in RL and GL **(G)** (n = 8). Rubisco LS and small subunit (SS) were separated in 12.5% SDS-PAGE, and the polypeptides were stained by Coomassie Brilliant Blue R-250 **(H)**. The membrane of the Western blot was stained by Ponceau S **(I)**, and the Western blotting showed LS in RL and GL **(J)**. Diameter of red (RS) and green stems (GS) **(K)** (n = 15) and dry weight of red and green *M. micrantha* plants **(L)** (n = 8). The error bars represent the standard errors (SEs) of 8 to 15 biological replicates, and the asterisks indicate significant differences (two-sided Student’s t-test, *P < 0.05, **P < 0.01, ***P < 0.001).

The previously discussed results showed that, compared with green *M. micrantha* plants, red plants could tolerate lower temperature. Both the accumulation of anthocyanins in leaves and low temperature are associated with photosynthetic capability (Hughes et al., 2007; Sun et al., 2018). Photosynthetic capability is the basis of the biomass accumulation of *M. micrantha*. Whether this species can accumulate additional biomass during winter is a reflection of its adaptability to low-temperature environments. Therefore, we compared indicators related to photosynthesis. Stomatal aperture directly affects leaf gas exchange. The stomata of *M. micrantha* leaves were comparatively examined by SEM. During winter, the stomata of green leaves were not fully open and most of them were even closed, while the stomata of red leaves were partially open. To understand their relevance with other physiological activities, the density and aperture of stomata were further estimated. The resulting data showed that the density and aperture of stomata on red leaves were higher and larger than those on green leaves (**Figures 3A and B**). Compared with that of green leaves, the stomatal aperture of red leaves is relatively large. The *G*_s_ parameters related to stomatal aperture showed that *G*_s_ were significantly greater in red leaves than in green leaves (**Figure 3E**). The results concerning the *P*_n_ and *T*_r_ were similar to those concerning *G*_s_, and all three parameters were significantly greater in the red leaves than in the green leaves (**Figures 3C, D**). In contrast, the *C*_i_ results were different from those of the other gas exchange parameters. The *C*_i_ of the green leaves was significantly greater than that of the red leaves (**Figure 3F**). The contents of Chl and Rubisco, both of which are related to photosynthesis, were relatively low in the green leaves (**Figures 3G, H**), which were consistent with the results of the *P*_n_. Western blotting analysis on Rubisco LS (**Figure 3J**) and the result of Western blot membrane stained by Ponceau S (**Figure 3I**) were consistent with SDS-PAGE analysis (**Figure 3H**). The stem diameter and biomass results showed that the diameter of the red stems was significantly greater than that of the green stems. The dry matter accumulation of the red *M. micrantha* plants during the same time period was also significantly greater than that of the green plants (**Figures 3K, L**).

## Discussion

As a highly invasive weed, *M. micrantha* is native to parts of South America that have a tropical, high-temperature environment. However, South China, one of the areas infested with *M. micrantha*, is a subtropical region with a low-temperature environment (below 15°C) during winter (Yan et al., 2013). To adapt to the low-temperature environment during winter, the leaves of *M. micrantha* plants exhibit a striking redness due to the presence of anthocyanins. Spectroscopic results show that the extracts from the stems and leaves of *M. micrantha* have an absorption peak at 530 nm, and the absorption peak of red *M. micrantha* plants is significantly higher than that of green *M. micrantha* plants. Anthocyanins are induced by a number of environmental factors, including high light and UV-B radiation and cold temperatures, and anthocyanins have been proposed to be important compounds involved in the abiotic stress tolerance response (Lorenc-Kukula et al., 2005; Zhang et al., 2016; Sivankalyani et al., 2016; Liu et al., 2018). There are two main functions of anthocyanins in plants: one involves photoprotection by functioning as a light screen, and the other involves antioxidation by scavenging ROS. The results of this study showed that anthocyanins in *M. micrantha* leaves were more likely to have antioxidant effects in winter.

Studies have shown that low temperatures can induce anthocyanin formation (Lo Piero et al., 2005; Li et al., 2017b; Carmona et al., 2017). During winter, a large amount of anthocyanins, total phenols, and flavonoids accumulated in red *M. micrantha* (**Figures 1C–E**). This result is consistent with that of woody plants during winter (Zhu et al., 2018). Low temperatures during winter lead to decreased fluidity of plant cell membranes, decreased activity of enzymes, oxidative stress, and the accumulation of ROS. As toxic substances, ROS can destroy biological macromolecules, attack cell membranes, and accelerate leaf damage. Anthocyanins, total phenols, and flavonoids can improve the antioxidant capability of plants. The results showed that the total antioxidant capability of the leaves and stems of red *M. micrantha* was significantly greater than that of green *M. micrantha*. A study of sweet potato showed that the hydrogen peroxide content in the leaves increased significantly and that membrane lipid peroxidation increased at 4°C (Jin et al., 2017). In the present study, red and green *M. micrantha* plants were placed in a 4°C incubator for 12 h. The results showed that more ROS (superoxide anions and hydrogen peroxide) accumulated in the stems and leaves of green *M. micrantha* than in red *M. micrantha*. This finding indicated that the accumulation of anthocyanins could effectively eliminate ROS under low-temperature stress.

To test the tolerance of the different phenotypes of *M. micrantha* to low-temperature stress, red and green *M. micrantha* plants were cultivated in a 4°C incubator. After 12 h, the *F*_v_/*F*_m_ was measured using a Chl fluorescence imaging system (Technologica, UK). The results showed that the *F*_v_/*F*_m_ value of the red leaves did not decrease significantly after low-temperature exposure; however, the *F*_v_/*F*_m_ value of the red stems, green leaves, and green stems decreased significantly. The greatest decrease in *F*_v_/*F*_m_ value was detected in green leaves and stems. The *F*_v_/*F*_m_ is an important parameter of Chl fluorescence and does not change significantly when a plant is under normal conditions. However, the *F*_v_/*F*_m_ will decrease significantly when a plant is under stress (drought, high light, low temperature, etc.) or is aging (Zhang et al., 2016; Guadagno et al., 2017; Jin et al., 2017; Yu et al., 2017; Sun et al., 2018). Thus, the results showed that anthocyanin accumulation could improve plant tolerance to low temperature.

Low temperatures limit photosynthesis in many plant species. As the only source of organic matter in higher plants, photosynthesis plays a decisive role in the accumulation of plant biomass. Low-temperature environments can reduce the activity of enzymes and affect the activity of Rubisco, which is related to photosynthesis, thus reducing the photosynthetic rate. Low temperature also decreases membrane fluidity, affects stomatal activity, and limits gas exchange. Comparing the *P*_n_ and *G*_s_, we found that the values in red leaves were significantly higher than those in green leaves. The *G*_s_ of plant leaves is closely related to stomatal opening and closing. Studies have shown that under drought stress, stomatal closure can reduce the *G*_s_ of leaves (Li et al., 2017; Takahashi et al., 2018). In the present study, SEM revealed that the stomatal aperture of the red leaves was larger than that of the green leaves. Photosynthesis involves “energy capture” (light reactions), in which light energy is converted to chemical energy, and “energy utilization” (carbon reactions), in which chemical energy is used to convert CO_2_ to carbohydrates. Chl and Rubisco proteins play key roles in these two reactions, and their contents directly affect photosynthesis. In this experiment, Chl and Rubisco proteins in red leaves were significantly higher than those in green leaves, which was consistent with the *P*_n_ results. It has been reported that Chl and Rubisco protein contents in leaves are low and that the photosynthetic system is fragile when plant leaves are not fully mature. To reduce photooxidative damage, anthocyanin contents in leaves are increased (Zhang et al., 2016). Other studies have shown that plants grown in high-light environments present increased anthocyanin contents to filter excess light energy. The main role of anthocyanins in both cases is to reduce the absorption of light by leaves, resulting in a decrease in the photosynthetic rate of plant leaves (Lev-Yadun and Gould, 2009). However, the *P*_n_ and Chl and Rubisco protein contents in this study were significantly greater in red leaves than those in green leaves. This finding shows that anthocyanin function mainly as an antioxidant, which is consistent with results reported in mango (Sivankalyani et al., 2016).

As an invasive plant, the adaptation strategy of *M. micrantha* to its environment is obviously to increase its invasiveness. *M. micrantha* is known as “mile-a-minute grass,” so its rapid growth is a reflection of its strong invasiveness. Biomass accumulation depends on the rapid growth of plants. The results show that the biomass of red *M. micrantha* is significantly greater than that of green *M. micrantha* during the same period of winter. Moreover, the stems of red *M. micrantha* are thicker than those of the green type. The accumulation of anthocyanins in *M. micrantha* under low temperature during winter resulted in improved antioxidant capability and reduced oxidative stress, increasing its adaptability to low-temperature environments and subsequently increasing its photosynthetic rate and biomass.

## Data Availability

All datasets for this study are included in the manuscript and/or the Supplementary Files.

## Author Contributions

QZ and JZ designed the experiments. LS and WL performed all experiments. QZ and JZ analyzed the data. QZ wrote the manuscript. CP revised the manuscript. All the authors approved the final version of the manuscript.

## Funding

The study was supported by the National Basic Research Program of China (2017YFC1200105) and the National Natural Science Foundation of China (31570398, 31870374). This work was also supported by Guangdong Province Natural Science Foundation (2017A030313167), and the study was also supported by the Innovation Project of Graduate School of South China Normal University.

## Conflict of Interest Statement

The authors declare that the research was conducted in the absence of any commercial or financial relationships that could be construed as a potential conflict of interest.
