# Corrigendum: Accumulation of Anthocyanins: An Adaptation Strategy of *Mikania micrantha* to Low Temperature in Winter

**DOI:** 10.3389/fpls.2019.01796

**Published:** 2020-02-04

**Authors:** Qilei Zhang, Junjie Zhai, Ling Shao, Wei Lin, Changlian Peng

**Affiliations:** ^1^ Guangzhou Key Laboratory of Subtropical Biodiversity and Biomonitoring, Guangdong Provincial Key Laboratory of Biotechnology for Plant Development, School of Life Sciences, South China Normal University, Guangzhou, China; ^2^ College of Life Science, Zhao Qing University, Zhaoqing, China

**Keywords:** anthocyanins, antioxidant activity, gas exchange, *Mikania micrantha*, winter

In the original article, there was a mistake in the legend for **Figure 3** as published. The mistake was in the first sentence “Electron microscopy” was used instead of “Stomatal density.” The correct legend appears below.

**Figure 3:**
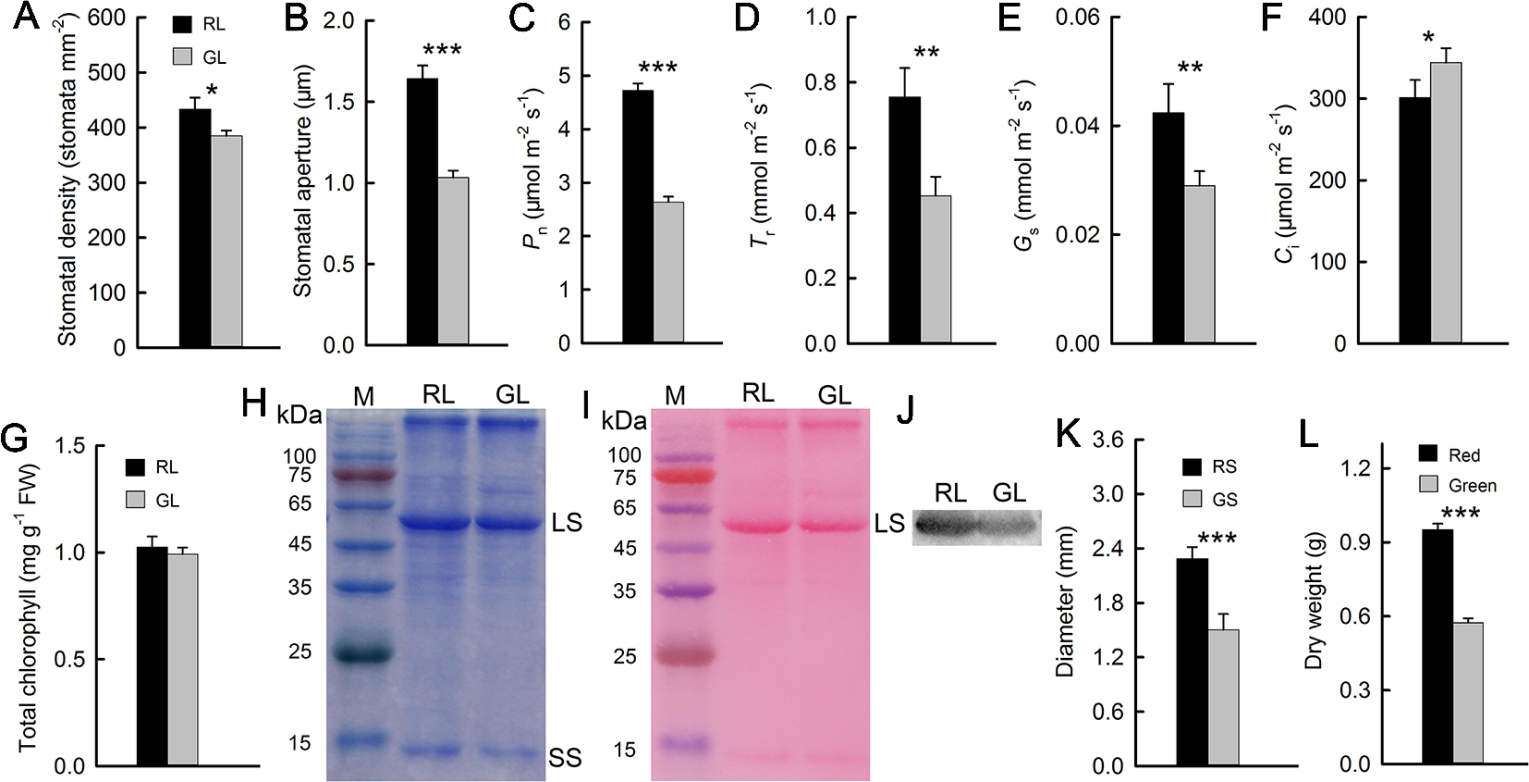
Stomatal density and aperture of red (RL) and green leaves (GL) **(A, B)**. Gas exchange parameters of red and green leaves, including the net photosynthetic rate (*P*_n_) **(C)**, transpiration rate (*T*_r_) **(D)**, stomatal conductance (*G*_s_) **(E)**, and intercellular CO_2_ concentration (*C*_i_) **(F)** (n = 10). Chlorophyll (Chl) content in RL and GL **(G)** (n = 8). Rubisco LS and small subunit (SS) were separated in 12.5% SDS-PAGE, and the polypeptides were stained by Coomassie Brilliant Blue R-250 **(H)**. The membrane of the Western blot was stained by Ponceau S **(I)**, and the Western blotting showed LS in RL and GL **(J)**. Diameter of red (RS) and green stems (GS) **(K)** (n = 15) and dry weight of red and green *M. micrantha* plants **(L)** (n = 8). The error bars represent the standard errors (SEs) of 8 to 15 biological replicates, and the asterisks indicate significant differences (two-sided Student’s *t*-test, *P < 0.05, **P < 0.01, ***P < 0.001).

Further, there was a mistake in **Figures 3A, B** as published. In **Figure 3**, two images (A and B) were provided to show stomata, which could not show a statistical relevance claimed in our context. Herein, we prefer to replace the two images with stomata density (A) and aperture size (B), which are statistically appropriate to show a relevance between stomata and photosynthetic rate (*P*_n_) (C), transpiration rate (*T*_r_) (D), stomatal conductance (*G*_s_) (E), or/and intercellular CO_2_ concentration (*C*_i_) (F). The corrected **Figure 3** appears below.

Additionally, there was an error in the results of our original publication. We incorrectly stated that “The stomata of *M. micrantha* leaves were viewed by SEM. During winter, the stomata of *M. micrantha* leaves were not fully open, and many of them were even closed (**Figures 3A, B**). Compared with that of green leaves, the stomatal aperture of red leaves is relatively large.”

A correction has been made to the **Results** section, subsection **Gas Exchange Parameters and Biomass**:

“The previously discussed results showed that, compared with green *M. micrantha* plants, red plants could tolerate lower temperature. Both the accumulation of anthocyanins in leaves and low temperature are associated with photosynthetic capability (Hughes et al., 2007; Sun et al., 2018). Photosynthetic capability is the basis of the biomass accumulation of *M. micrantha*. Whether this species can accumulate additional biomass during winter is a reflection of its adaptability to low-temperature environments. Therefore, we compared indicators related to photosynthesis. Stomatal aperture directly affects leaf gas exchange. The stomata of *M. micrantha* leaves were comparatively examined by SEM. During winter, the stomata of green leaves were not fully open and most of them were even closed, while the stomata of red leaves were partially open. To understand their relevance with other physiological activities, the density and aperture of stomata were further estimated. The resulting data showed that the density and aperture of stomata on red leaves were higher and larger than those on green leaves (**Figures 3A and B**). Compared with that of green leaves, the stomatal aperture of red leaves is relatively large. The *G*_s_ parameters related to stomatal aperture showed that *G*_s_ were significantly greater in red leaves than in green leaves (**Figure 3E**). The results concerning the *P*_n_ and *T*_r_ were similar to those concerning *G*_s_, and all three parameters were significantly greater in the red leaves than in the green leaves (**Figures 3C, D**). In contrast, the *C*_i_ results were different from those of the other gas exchange parameters. The *C*_i_ of the green leaves was significantly greater than that of the red leaves (**Figure 3F**). The contents of Chl and Rubisco, both of which are related to photosynthesis, were relatively low in the green leaves (**Figures 3G, H**), which were consistent with the results of the *P*_n_. Western blotting analysis on Rubisco LS (**Figure 3J**) and the result of Western blot membrane stained by Ponceau S (**Figure 3I**) were consistent with SDS-PAGE analysis (**Figure 3H**). The stem diameter and biomass results showed that the diameter of the red stems was significantly greater than that of the green stems. The dry matter accumulation of the red *M. micrantha* plants during the same time period was also significantly greater than that of the green plants (**Figures 3K, L**).”

The authors apologize for these errors and state that these does not change the scientific conclusions of the article in any way. The original article has been updated.
